# Hepatic Squamous Cell Carcinoma Diagnosed by Endoscopic Ultrasound-Guided Fine-Needle Aspiration

**DOI:** 10.1155/2021/9939898

**Published:** 2021-07-05

**Authors:** Koki Yamada, Susumu Shinoura, Kaoru Kikuchi

**Affiliations:** ^1^Department of Gastroenterology, Okinawa Chubu Hospital, 271 Miyazato, Uruma 904-2293, Japan; ^2^School of Psychology and Healthcare Management at Akasaka, Department of Healthcare Management, International University of Health and Welfare, 4-1-26 Akasaka, Minato, Tokyo 107-8402, Japan; ^3^Division of Gastroenterology, Yaeyama Hospital, 584-1 Maezato, Ishigaki 907-0002, Okinawa, Japan

## Abstract

Primary hepatic squamous cell carcinoma (SCC) is a rare malignancy with aggressive clinical features. This is the first case report of a primary hepatic SCC diagnosed by endoscopic ultrasound-guided fine-needle aspiration (EUS-FNA), which is a reliable and safe procedure for the histopathological diagnosis of liver lesions, even if the percutaneous approach is difficult due to ascites or hypervascularity at the puncture site. A 52-year-old man presented to the emergency department of a tertiary referral hospital with right upper quadrant abdominal pain and abdominal distention. Given the laboratory data, a diagnosis of spontaneous bacterial peritonitis (SBP) was made. Concurrently, an abdominal computed tomography (CT) scan revealed an 8 cm hypodense mass with delayed peripheral enhancement in the left hepatic lobe and paraaortic and perihepatic lymphadenopathy. As persistent ascites precluded percutaneous liver biopsy, we performed EUS-FNA of the liver mass, and the obtained specimen showed SCC. As otorhinolaryngological consultation and whole-body investigations, including chest CT, upper and lower endoscopy, and positron emission tomography CT, were all unremarkable except for the liver lesion and lymph nodes, a diagnosis of primary hepatic SCC with systemic lymph node metastasis was made. After treatment of SBP with antibiotics, we initiated chemotherapy concurrent with radiation therapy, adapted to his liver function. Radiation and three cycles of chemotherapy were not effective as the disease progressed, as seen on the follow-up CT scan, and the patient died of hepatic failure on the 134th day after diagnosis. In conclusion, EUS-FNA was a reliable method for tissue sampling in liver malignancies, particularly in selected patients with contraindications for percutaneous biopsy.

## 1. Introduction

Histopathologic examination of biopsy specimens obtained from atypical liver masses is known to provide essential diagnostic information, thus significantly improving patient care [1]. Occasionally, percutaneous liver biopsy is limited by ascites or coagulopathy secondary to poor liver function. Meanwhile, endoscopic ultrasound-guided fine-needle aspiration (EUS-FNA) has been a reliable method for diagnosing intraabdominal masses [2]. Multiple articles regarding EUS-FNA of the liver have been published, and according to the studies, EUS-FNA has had equivalent efficacy and safety compared with percutaneous liver biopsy [1]. This is the first case report of a hepatic squamous cell carcinoma (SCC) diagnosed by EUS-FNA in a patient with hepatic cirrhosis.

## 2. Case Presentation

**Figure 1:**
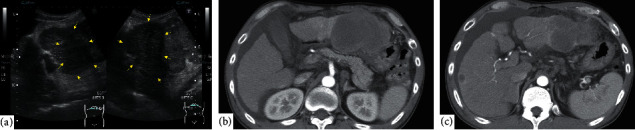
(a) Abdominal ultrasound showing a hypoechoic, mosaic-like, giant mass in the lateral segment of the left hepatic lobe of the cirrhotic liver (arrows). (b), (c) Early arterial phase of a contrast-enhanced computed tomography scan showing an 8 cm hypodense mass in the left hepatic lobe, a hypodense mass with ring-like enhancement in the right lobe, and multiple swollen lymph nodes.

**Figure 2:**
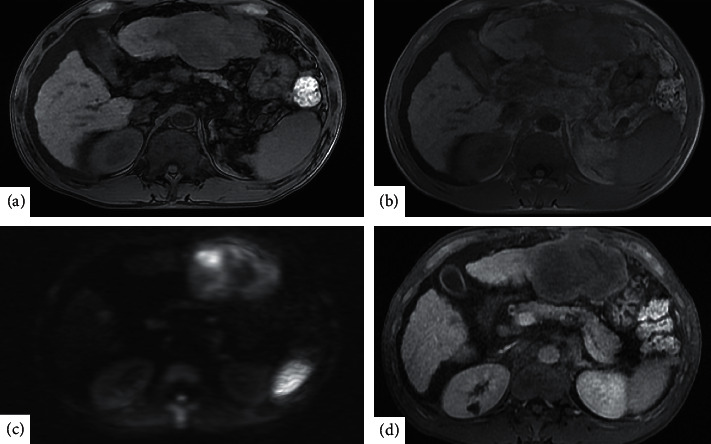
Marked hyperintensity on both the left and right hepatic lobe masses is revealed on diffusion-weighted magnetic resonance imaging (MRI), while peripheral arterial enhancement is seen on gadolinium ethoxybenzyl diethylenetriamine pentaacetic acid-enhanced MRI. (a) T1 in-phase. (b) T1 out-phase. (c) Diffusion-weighted image. (d) Hepatic phase.

**Figure 3:**
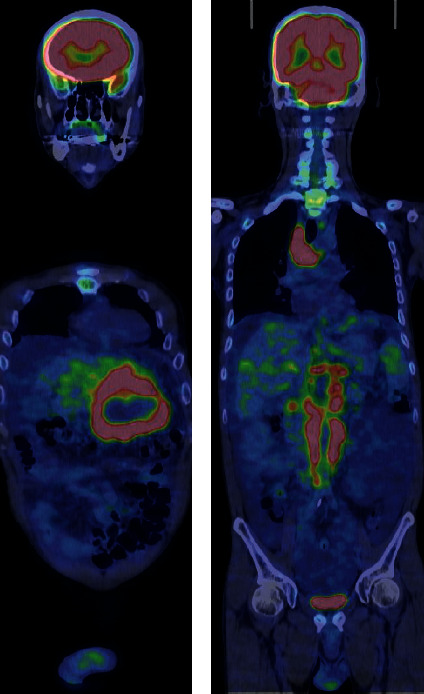
Fluorodeoxyglucose positron emission tomography scan showing significant accumulation in the left lobe and multiple hot spots in the paraaortic region.

A 52-year-old Japanese man with compensated liver cirrhosis secondary to hepatitis B presented to the emergency room of a tertiary referral hospital with chief complaints of fever, right upper quadrant abdominal pain, and abdominal distension. The patient had gained 4 kgs over the past 3 months. Before arriving at the emergency room, the patient had an episode of chills. His vital signs were as follows: blood pressure, 90/50 mmHg; pulse rate, 136 beats per minute; and body temperature, 39.4°C. On physical examination, the abdomen was distended with hepatomegaly and was positive for shifting dullness and tenderness in the left upper quadrant. Laboratory data were as follows: white blood cell count, 19800/*μ*L; platelet count, 92,000/*μ*L; alkaline phosphatase, 358 U/L; serum albumin, 2.6 g/dL; prothrombin time, 14.6 s; international normalized ratio, 1.27; alpha-fetoprotein, 19.5 ng/mL; carbohydrate antigen, 19-9, 132 U/ml; and protein induced by vitamin K absence-II of 16%. Ascitic fluid analysis showed a white cell count of 10,800/*μ*L (56% neutrophils). Blood and ascitic fluid cultures were negative. With regard to imaging studies, an abdominal ultrasound showed a hypoechoic, mosaic-patterned mass in the lateral segment of the left lobe of the cirrhotic liver (Figure 1(a)). A computed tomography (CT) scan with intravenous (IV) contrast revealed hypodense hepatic masses with peripheral delayed enhancement. The masses in the left and right hepatic lobes measured 8 cm and 2 cm, respectively. The CT scan also showed moderate ascites and paraaortic and perihepatic lymphadenopathy (Figure 1(b)). Marked hyperintensity of both the left and right hepatic lobe masses was revealed on diffusion-weighted magnetic resonance imaging (MRI), while peripheral arterial enhancement was seen on gadolinium ethoxybenzyl diethylenetriamine pentaacetic acid-enhanced MRI (Figures 2(a)–2(d)). A fluorodeoxyglucose-positron emission tomography (FDG-PET) scan showed significant accumulation in the left lobe and multiple hot spots in the paraaortic region (Figure 3).

**Figure 4:**
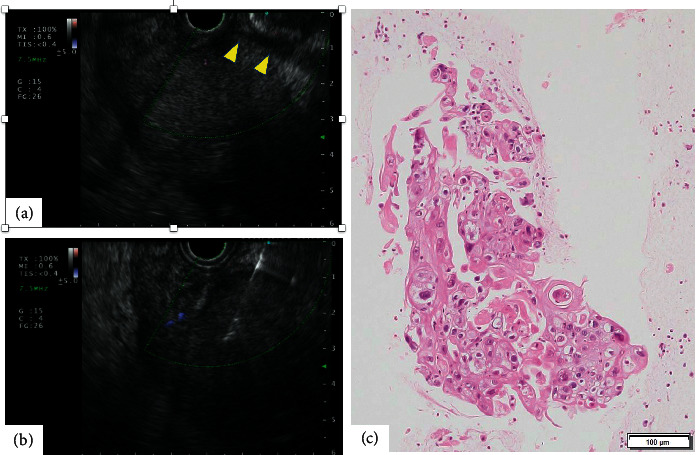
(a) Endoscopic ultrasound view from the gastric station showing no significant amount of ascites. Arrowheads correspond to the surface of an exposed hepatic mass. (b) Endoscopic ultrasound-guided fine-needle aspiration of the liver mass performed from the view without worrisome vasculature or ascites. (c) Histopathological view of squamous cell carcinoma with hematoxylin and eosin staining. Components of neither hepatocellular carcinoma nor adenocarcinoma are found.

Given the above laboratory data and imaging findings, a diagnosis of spontaneous bacterial peritonitis (SBP) and bilateral hepatic lobe lesion with multiple lymph node metastases was made. For SBP, IV antibiotics (ceftriaxone 2 g/day) were started. Based on the imaging findings, the radiologist suspected the formation of mixed type hepatocellular carcinoma or intrahepatic cholangiocarcinoma mass. On transabdominal ultrasound, the hepatic lesion was exposed at the hepatic surface without intervening normal hepatic component and ascites was noted at the hepatic surface. These factors precluded percutaneous liver mass biopsy because of the high risk of bleeding. On the other hand, EUS view from the gastric station showed no significant amount of ascites (Figure 4(a)). EUS-FNA targeting the left lobe mass was performed from the gastric station view without worrisome vasculature or ascites, by using a 22-gauge aspiration needle (EXPECT®, Boston Scientific Co., Natick, MA, USA) with 3 passes (Figure 4(b)). Though rapid on-site evaluation was not performed due to the shortage of a pathology technician, an adequate amount of the specimen was obtained without any complications. The histopathological analysis showed a well-differentiated squamous cell carcinoma with necrosis. Components of neither hepatocellular carcinoma nor adenocarcinoma were found in the specimen. Finally, a pathological diagnosis of SCC was made (Figure 4(c)).

As we initially suspected metastatic SCC of the liver, an extensive workup including chest CT, esophagogastroduodenoscopy, colonoscopy, and otorhinolaryngological examination was performed, all of which showed negative findings. Given the above results, a diagnosis of primary SCC of the liver was made.

After 2 weeks of antibiotic treatment for SBP, the patient's liver function gradually improved to a Child–Pugh's score of 6. The patient was fully informed that the diagnosis was primary SCC of the liver and was in agreement with the adjusted chemoradiation therapy. The patient was started on radiation therapy (50 Gy) for the liver mass and paraaortic lymph nodes, followed by chemotherapy comprising cisplatin (50 mg/m^2^) and fluorouracil (500 mg/m^2^). Given the patient's comorbidity of cirrhosis, the dose of the chemotherapy was reduced. In total, three cycles of chemotherapy were administered. Radiation with three cycles of chemotherapy was not effective as the disease progressed, as seen on the follow-up CT scan, and the patient died of hepatic failure on the 134th day after diagnosis.

Written informed consent was obtained from the patient's family for the publication of this case report and its accompanying images. This study was also approved by the hospital board committee (approval number OCH-66).

## 3. Discussion

Primary hepatic SCC is rare. Zhang et al. [3] enlisted 31 cases that have been previously reported in the literature and four other patients encountered in their practice. According to the article [3], the average age of the patients was 54 years (range of 18–83 years) with a male to female ratio of 19 : 16. Zhang et al. [3] discussed that of the above 35 patients, 57% (20/35) had liver cysts, 20% (7/35) had bile duct stones, and 5.7% (2/35) had both with a tendency of worse prognosis in SCC patients with accompanying liver cysts than with bile duct stones. Other possible risk factors for primary SCC of the liver were hepatic teratoma, liver cirrhosis, and Caroli's disease [4]. A possible explanation for the occurrence of primary SCC of the liver is a hypothesis—the lesion may have originated from the tumor transformation of the biliary epithelium under chronic inflammation or metaplasia and subsequent neoplastic transformation of the preexisting cysts of the liver [3]. However, no proven explanation for the mechanism is available thus far. The clinical course of primary hepatic SCC, especially with underlying liver cirrhosis, is very aggressive, and it is difficult to achieve survival of >6 months even with several therapies combined (including surgical resection, chemotherapy, radiotherapy, and transcatheter arterial chemoembolization) [5]. The most frequently used chemotherapy regimen for hepatic SCC as described in the literature comprises cisplatin and fluorouracil [6]. However, due to its rarity, the definite management of hepatic SCC has not been established thus far.

Percutaneous liver biopsy remains an essential tool for the evaluation of focal liver masses [7]. Sensitivity, positive predictive value (PPV), negative predictive value (NPV), and overall accuracy were 90%, 100%, 51.7%, and 91%, respectively [8, 9]. Moreover, a percutaneous approach can target multiple parts of the liver including the bilateral and caudate lobes.

The contraindications of percutaneous liver biopsy are thrombocytopenia and coagulopathy [10]. It is suggested that platelet counts of less than 50,000/mm^3^ or a PT-INR of above 1.5 should force clinicians to consider suspending the procedure to avoid bleeding [7]. With regard to bleeding complications, the rate is reported to be around 2% [11]. Additionally, a systematic review of 1340 patients evaluated the risk of needle tract seeding following percutaneous liver tumor biopsy, which was reported to be 2.7% overall [12].

In contrast, a prospective study reported the diagnostic accuracy of EUS-FNA in hepatic masses to be between 82% and 100% and the sensitivity, specificity, NPV, and PPV to be 94%, 100%, 78%, and 100%, respectively [13]. Additionally, EUS-FNA can produce specimens at the most comparable to the specimens obtained in percutaneous liver biopsy [13]. One study that directly compared percutaneous liver biopsy (60 patients) and EUS-FNA (30 patients) revealed that EUS-FNA has equivalent feasibility of histological diagnosis (percutaneous liver biopsy = 100% and EUS-FNA = 93%, *p*=0.841).

If the liver tissue sampling is aimed at a “benign-malignant assessment,” a 22-gauge aspiration needle can obtain an adequate specimen for diagnosis [2, 13]. However, to visualize and target the right lobe mass with EUS-FNA is actually difficult [14], and it is suggested that percutaneous biopsy would be a better option for lesions that are located in the right lobe of the liver. When concerned about adverse events, EUS-FNA appears to be a safe and minimally invasive procedure for the normal liver, with an almost equivalent or even less bleeding complication rate as compared with percutaneous tumor biopsy (of less than 2%) [15, 16]. To the best of our knowledge, only one case of needle-track seeding following EUS-FNA of the liver was reported [17].

In the present case, we decided to perform EUS-FNA as the liver masses were mainly located in the left lobe and the visibility from the gastric station on EUS provided us with an excellent view of the liver mass without worrisome vasculature or ascites as compared to the percutaneous view. Based on clinical studies which showed that both 19-gauge and 22-gauge needles attain equivalent feasibility in histological diagnosis [15] and that it is preferable to choose a thinner needle to reduce bleeding complications [18], we chose a 22-gauge needle instead of a 19-gauge to obtain the specimen to minimize the risk of bleeding. An adequate quantity of the specimen was obtained for diagnosing malignancy, and no adverse events were observed after EUS-FNA in this case.

Given the rarity of the primary hepatic SCC, adenosquamous cell carcinoma was a differential diagnosis in the present case; it was less likely from the histopathological evaluation that the specimen was negative for individual cell keratinization, intracellular bridges, glandular differentiation, or intracellular and intraluminal mucin accumulation, which were the characteristic features of adenosquamous cell carcinoma [19].

Primary hepatic SCC is rare and is known for its aggressive clinical course. We concluded that EUS-guided fine-needle biopsy was a reliable method for tissue sampling in liver malignancies, particularly in selected patients with contraindications for percutaneous biopsy.

## Data Availability

Data cannot be shared publicly because the patient could be imaginable from the clinical dataset including age, sex, visiting date, and period of admission. Data are available from the Okinawa Chubu Hospital Ethics Committee (contact via the study protocol (OCH-66) https://chubuweb.hosp.pref.okinawa.jp/rinshou/koukai/) for researchers who meet the criteria for access to confidential data.
